# Reactive Oxygen Species Link Gene Regulatory Networks During *Arabidopsis* Root Development

**DOI:** 10.3389/fpls.2021.660274

**Published:** 2021-04-27

**Authors:** Kosuke Mase, Hironaka Tsukagoshi

**Affiliations:** Faculty of Agriculture, Meijo University, Nagoya, Japan

**Keywords:** reactive oxygen species, *Arabidopsis thaliana*, transcription factor, primary root development, stem cell niche, lateral root development, root hair development, crosstalk

## Abstract

Plant development under altered nutritional status and environmental conditions and during attack from invaders is highly regulated by plant hormones at the molecular level by various signaling pathways. Previously, reactive oxygen species (ROS) were believed to be harmful as they cause oxidative damage to cells; however, in the last decade, the essential role of ROS as signaling molecules regulating plant growth has been revealed. Plant roots accumulate relatively high levels of ROS, and thus, maintaining ROS homeostasis, which has been shown to regulate the balance between cell proliferation and differentiation at the root tip, is important for proper root growth. However, when the balance is disturbed, plants are unable to respond to the changes in the surrounding conditions and cannot grow and survive. Moreover, ROS control cell expansion and cell differentiation processes such as root hair formation and lateral root development. In these processes, the transcription factor-mediated gene expression network is important downstream of ROS. Although ROS can independently regulate root growth to some extent, a complex crosstalk occurs between ROS and other signaling molecules. Hormone signals are known to regulate root growth, and ROS are thought to merge with these signals. In fact, the crosstalk between ROS and these hormones has been elucidated, and the central transcription factors that act as a hub between these signals have been identified. In addition, ROS are known to act as important signaling factors in plant immune responses; however, how they also regulate plant growth is not clear. Recent studies have strongly indicated that ROS link these two events. In this review, we describe and discuss the role of ROS signaling in root development, with a particular focus on transcriptional regulation. We also summarize the crosstalk with other signals and discuss the importance of ROS as signaling molecules for plant root development.

## Introduction

Oxygen (O_2_) is a stable molecule that is required for the survival of aerobic organisms on Earth. However, during various *in vivo* processes, especially respiration, it can change into high-energy molecules called reactive oxygen species (ROS; superoxide, O_2_^–^; hydrogen peroxide, H_2_O_2_; hydroxyl radical, ^⋅^OH; and singlet oxygen, ^1^O_2_). In general, high levels of ROS are known to be cytotoxic because their oxidative properties in living cells cause damage to DNA, lipids, and proteins.

In plants, ROS are produced as a by-product of normal aerobic metabolism processes such as electron transport chains or redox reactions in chloroplasts or mitochondria (Hossain et al., 2015). Even other cellular compartments such as peroxisomes and microsomes generate ROS (Hossain et al., 2015). NADPH oxidases (RESPIRATORY BURST OXIDASE HOMOLOG proteins, also called RBOH proteins), which are known as O_2_^–^ generators, function in fundamental respiration processes such as photosynthetic electron transport chains and mitochondrial respiratory electron transport chains as well as play a role in catalyzing ROS on the plasma membrane (Chapman et al., 2019; Kaya et al., 2019). O_2_^–^, a precursor of various ROS, is converted to H_2_O_2_ spontaneously or enzymatically *via* superoxide dismutase (SOD; Wang Y. et al., 2018), oxalate oxidase (Caliskan and Cuming, 1998), or diamine oxidase (Federico and Angelini, 1986). ROS are generated both inside and outside cells, although their lifespan is very short. Among ROS, H_2_O_2_ is the most stable (half-life, more than 1 ms), is considered an important redox signaling molecule (Mhamdi and Van Breusegem, 2018), and is spontaneously metabolized to H_2_O and O_2_ by class III peroxidases (Smirnoff and Arnaud, 2019). ^⋅^OH possesses the highest oxidizing power and is unstable (half-life, 1 ns; Mittler, 2017; Mhamdi and Van Breusegem, 2018). ^1^O_2_ is usually formed in chloroplast photosystem II (Dmitrieva et al., 2020). Although ROS are cytotoxic, previous studies have shown that plants utilize them as signaling molecules to develop organs and respond to stress by regulating gene expression.

Plants are constantly subjected to various abiotic and biotic stresses such as salt, drought, and pathogen attack, which significantly increase ROS levels, leading to redox imbalance in their life cycle (Hasanuzzaman et al., 2020). Plants also accumulate massive amounts of ROS for protection against pathogen attack, which involves cell death of plant cells themselves (Qi et al., 2017). The excess accumulation of ROS might damage their organs and could occasionally lead to dysfunction or death. However, appropriate ROS levels act as signaling molecules for organ development and in response to biotic or abiotic stress. In fact, plant root tips constantly accumulate ROS levels that are not found in normal leaves (Dunand et al., 2007). In the process of evolution, plants have established an elaborate system for controlling oxidative stress and using ROS as signaling molecules. Herein, we introduce the important role of ROS homeostasis, focusing on plant roots.

Roots play critical roles in plants. They structurally support the plant and provide water and nutrients for survival. Furthermore, roots act as sensors for detecting alterations in the surrounding environment, such as drought and salt stress and presence of microorganisms. Roots also maintain growth in the direction of gravity with cell proliferation and differentiation in the root tip. In addition, lateral roots and root hairs facilitate the expansion of the surface and rhizosphere. Plant roots can be distinguished into different zones from the root tip to the base along the longitudinal axis based on their characteristics: the meristematic, elongation, and maturation zones (Petricka et al., 2012).

Root growth occurs by the repeated cell division in the meristematic zone. A quiescent center (QC) is located in the apical meristem, which hardly divides, but is surrounded by a stem cell niche (SCN). The major genetic regulators of root growth include the AP-2 transcription factor PLETHORAs (PLTs; Aida et al., 2004), the GRAS family transcription factor SHORT ROOT (SHR; Benfey et al., 1993; Helariutta et al., 2000), SCARECROW (SCR; Di Laurenzio et al., 1996; Sabatini et al., 2003), and the homeodomain transcription factor WUSCHEL-related homeobox 5 (WOX5; Sarkar et al., 2007). Both PLT1 and PLT2 are required for distal cell division and stem cell maintenance (Aida et al., 2004), and redox balance has been reported to affect PLT functions (Licausi et al., 2013). SHR is first expressed in the stele (Benfey et al., 1993; Helariutta et al., 2000) and then moves into the cells, including the QC and endodermis (Nakajima et al., 2001). SCR, which is one of the downstream SHR transcription factors, plays a role in radial patterning and acts cell-autonomously for the distal specification of the QC interacting with SHR at the protein level (Di Laurenzio et al., 1996; Nakajima et al., 2001; Sabatini et al., 2003; Levesque et al., 2006) in a parallel pathway to PLT-related network (Aida et al., 2004). SCR–SHR regulates asymmetric cell division at the SCN and determines the identity of the endodermis and cortex cells by regulating the expression of cell cycle-related genes (Nakajima et al., 2001; Sozzani et al., 2010). WOX5, which is expressed at the QC, acts non-cell-autonomously to prevent stem cell differentiation downstream of SHR and SCR, but not of PLT proteins (Sarkar et al., 2007). In addition to these transcription factors, many other factors generate complex gene networks and systems for the maintenance of the SCN (Iyer-Pascuzzi and Benfey, 2009; Petricka et al., 2012). Thus, a gene regulatory network controlled by several key transcription factors ensures proper root development by controlling cell patterning in the SCN.

In the elongation zone, cells stop proliferating and rapidly begin to elongate along the longitudinal axis with cell wall loosening. The primary plant cell wall is composed of several polysaccharides such as cellulose, hemicelluloses, and pectins (Gorshkova et al., 2013). Although cell wall enzymes generate and modify cell wall components, ROS are also important for cell wall remodeling (hardening or loosening). Many studies have shown that ROS generated in the apoplast *via* NADPH oxidases in the plasma membrane are involved in controlling cell wall rigidity (Kärkönen and Kuchitsu, 2015). As mentioned above, extracellular O_2_^–^ is spontaneously or enzymatically converted to H_2_O_2_ and then to ^⋅^OH. The release of these reactive oxygen radicals can enzyme-independently oxidize cell wall polysaccharides *via* electron transfer (Kärkönen and Kuchitsu, 2015; Somssich et al., 2016). Especially, ^⋅^OH cleaves pectin and/or hemicellulose resulting in the loosening of the cell wall in the elongation zone (Chen and Schopfer, 1999). Conversely, the accumulation of apoplastic H_2_O_2_ and ROS scavengers such as ascorbic acid can inhibit cell wall elongation (Somssich et al., 2016). Thus, during cell wall remodeling, apoplastic ROS homeostasis helps cells in the elongation zone to control vertical growth.

In the maturation zone, cell maturation involves finely differentiated organs such as the Casparian strip, root hair, and lateral root. The Casparian strip acts as a diffusion barrier in the root endodermal cell layers of vascular plants and helps to protect against pathogen attack and to conduct selective nutrient uptake (Barberon, 2017; Nakamura and Grebe, 2018). The establishment of the Casparian strip in *Arabidopsis* roots requires localized ROS production. Several NADPH oxidases—mainly RBOHF and RBOHD—and peroxidases produce ROS in the extracellular matrix through the action of localized Casparian strip domain proteins (CASPs), which act as scaffolds on the root endodermal plasma membrane (Fujita et al., 2020). During Casparian strip formation, localized ROS production facilitates oxidative polymerization of monolignols to form lignin macromolecules (Liu, 2012). Casparian strip formation is initiated by the binding of the vasculature-derived peptide, Casparian strip integrity factor (CIFs), and its receptor, SCHENGEN3 (SGN3), which colocalizes on the endodermis with SGN1 kinase (Fujita et al., 2020), followed by the expression of transcription factors such as MYB36 (Kamiya et al., 2015), MYB41 (Kosma et al., 2014), and MYB15 (Chezem et al., 2017), which regulate Casparian strip formation, suberization, and lignification, respectively, by regulating downstream gene expression (Fujita et al., 2020). In particular, MYB36 directly and positively regulates the expression of genes required for Casparian strip formation, such as *CASP1*, *PEROXIDASE 64* (*PER64*), and *Enhanced Suberin 1* (*ESB1*; Kamiya et al., 2015). Thus, under these signaling pathways, local ROS accumulation around the root endodermal cells contributes to lignification, which acts as a diffusion barrier. Conversely, root hair and lateral root play important roles in the expansion of the rhizosphere. ROS are also involved as signaling molecules in this process.

Although ROS are known to regulate various aspects of root development, they themselves do not control root growth. This process is controlled by a very complex signal network, which especially involves interaction with plant hormones. Auxin, which is one of the critical plant hormones, has physiological activity and significantly affects several plant development processes such as cell proliferation and elongation. In the root, auxin accumulates at high concentration in the apical meristem and forms a gradient that decreases toward the basal meristem according to polar transport, which is mainly regulated by auxin influx and efflux carriers—AUX and PINs—respectively (Armengot et al., 2016). In addition, cytokinin, which is an antagonist hormone of auxin, is important and affects plant development along with auxin activity. Morphological formation has been shown to be controlled by auxin and cytokinin signaling; moreover, redox balance is known to play an important role in hormonal activity (Tognetti et al., 2017). Other hormones such as brassinosteroids (BRs), abscisic acid (ABA), and salicylic acid (SA) are also involved in ROS signaling (Overmyer et al., 2003; Lv et al., 2018; Planas-Riverola et al., 2019). In the crosstalk between ROS and plant hormones, various key transcription factors and downstream secondary messengers have been found to play important roles.

To prove that ROS act as signaling molecules, as described above, it is important to detect ROS at the cellular level. Therefore, accurate and specific tools for detecting each ROS are required. For instance, nitroblue tetrazolium (NBT) and diaminobenzidine (DAB) are widely used for the classical staining methods. NBT can be used to detect or quantify O_2_^–^ by observing blue deposits under a bright field or by formazan extraction (Driever et al., 2009; Bournonville and Díaz-Ricci, 2011). DAB reacts with H_2_O_2_ to form brown polymerization products (Driever et al., 2009). Since DAB staining is performed under relatively low pH conditions (pH < 3.6), care should be taken to avoid ROS production under these experimental conditions rather than DAB staining itself (Swanson et al., 2011). DAB shows relatively low specificity for ROS. Both staining methods can detect ROS at the tissue level, but they cause cell death. To measure the real-time behavior of ROS, more accurate and live-cell methods using fluorescent probes such as dihydroethidium (DHE), hydroxyphenyl fluorescein (HPF), 2′-7′-dichlorodihydrofluorescein diacetate (DCFH-DA), H_2_O_2_-3′-O-acetyl-6′-O-pentafluorobenzenesulfonyl-2′-7′-difluorofluorescein-Ac (H_2_O_2_-BES-Ac), and peroxy orange 1 (PO1) have been developed. DHE produces red fluorescence and is used to detect O_2_^–^ (Benov et al., 1998; Kalyanaraman et al., 2012). HPF can detect highly reactive oxygen species such as ^⋅^OH and ONOO^–^, whereas it barely reacts with O_2_^–^, H_2_O_2_, and ^1^O_2_ (Setsukinai et al., 2003). DCFH-DA is frequently used to detect H_2_O_2_. A specific indicator for H_2_O_2_, H_2_O_2_-BES-Ac is useful for measuring intracellular H_2_O_2_ (Maeda et al., 2004; Maeda, 2008). PO1 produces intracellular fluorescence in response to H_2_O_2_ (Chang et al., 2004; Miller et al., 2005; Dickinson et al., 2010). However, because aromatic boronate-based indicators can react with ONOO^–^ faster than with H_2_O_2_, they may reduce the specificity for H_2_O_2_ detection (Ortega-Villasante et al., 2018). Furthermore, it is also possible to perform spatial detection of ROS by double staining with different ROS-specific probes with different fluorescence wavelength, for example DHE and BES-H_2_O_2_-Ac (Tsukagoshi et al., 2010). Additionally, promoter reporter constructs such as *pZat12:Luciferase* and *pWRKY40:Luciferase*, carrying the promoters of ROS-responsive genes, are utilized for live imaging in *Arabidopsis* for detecting the alterations in ROS levels indirectly through gene expression (Miller et al., 2009; Devireddy et al., 2018). Furthermore, HyPer, a transgenic fluorescent indicator, can be used for detecting intracellular H_2_O_2_ (Belousov et al., 2006). HyPer consists of a circularly permuted yellow fluorescent protein (cpYFP) inserted into OxyR-RD, which is the regulatory domain of an *Escherichia coli* peroxide sensor. Notably, the fluorescence of HyPer is pH dependent (Belousov et al., 2006; Swanson et al., 2011). Using these methods, it is possible to visualize the speed of spread and spatiotemporal regulation of ROS signaling in a live plant (Hernández-Barrera et al., 2015; Fichman et al., 2019). Many studies related to ROS require different methods for detecting and quantifying their levels at the scale of cells, tissues, and organs. These methods of ROS detection have led to the discovery of changes in ROS levels related to root growth and crosstalk with other signals.

Since many recent studies have revealed the molecular mechanisms regulated by ROS, we focused on the functions of ROS as signaling molecules in root development and on the crosstalk with other signals such as phytohormones and biotic stresses in *Arabidopsis thaliana*. In particular, we describe the regulation of gene expression by transcription factors, which act as key regulators of signal transduction.

## Ros Act as Signaling Molecules for Regulating Root Growth

The gradient distribution of ROS regulates the transition of cells from proliferation to differentiation at the root tip. O_2_^–^ accumulates in the meristematic zone, whereas H_2_O_2_ mainly accumulates in the elongation zone (Dunand et al., 2007). During ROS signaling, transcriptional control is one of the key regulators of root development. For maintaining the ROS balance between the meristematic and elongation zones, *UPBEAT1* (*UPB1*), which is a basic helix-loop-helix (bHLH) transcription factor (Tsukagoshi et al., 2010), plays a key role in the transcriptional regulation of ROS. UPB1 regulates ROS (H_2_O_2_ and O_2_^–^) homeostasis by repressing the expression of class III peroxidases in the elongation zone (Tsukagoshi et al., 2010). The changes in the spatial distribution of ROS alter the size of the meristematic zone. The *upb1-1* mutant has a larger meristematic zone than that of the wild type owing to the increase in the number of cells, which accumulates O_2_^–^, because the expression of UPB1-targeted peroxidases is not suppressed by UPB1. Thus, the root length of the *upb1-1* mutant is longer than that of the wild type. In contrast, the *UPB1* overexpression line has a reduced level of O_2_^–^ in the meristematic zone; thus, the size of the meristematic zone and the length of the root are smaller and shorter, respectively, than those of the wild type. These results indicate that the spatial distribution of at least two ROS, O_2_^–^ and H_2_O_2_, is critical for determining the cell status between proliferation and differentiation at the root tip (Tsukagoshi et al., 2010).

Root meristem growth factor 1 (RGF1) also controls root meristem size through ROS signaling (Matsuzaki et al., 2010; Yamada et al., 2020). RGF1 is an essential peptide hormone that controls the size of the meristematic zone, both as an intrinsic and extrinsic signal (Matsuzaki et al., 2010; Meng et al., 2012; Whitford et al., 2012; Yamada et al., 2020). Exogenous RGF1 treatment increases the size of the meristematic zone, whereas the *rgf1/2/3* triple mutant has a smaller meristematic zone (Matsuzaki et al., 2010). The H_2_O_2_ levels decreased and O_2_^–^ levels increased in the meristematic and elongation zones 24 h after treatment with the RGF1 peptide. In the RGF1 receptor mutant *rgfr1/2/3*, the levels of H_2_O_2_ and O_2_^–^ in the meristematic zone remained unchanged after RGF1 peptide treatment compared with those in the wild type. These data indicate that the RGF1-receptor pathway controls the distribution of ROS during the development of the root meristem (Yamada et al., 2020). In addition, *RGF1 INDUCIBLE TRANSCRIPTION FACTOR 1* (*RITF1*) was identified as a downstream factor in the RGF1–ROS signaling pathway (Yamada et al., 2020). The root meristem size was smaller and root growth rate was lower in the *ritf1* mutant than in the wild type. Furthermore, after RGF1 treatment, O_2_^–^ accumulation was lower in the *ritf1* mutant than in the wild type (Yamada et al., 2020). In addition, ROS signals modulated by RITF1 regulate the stability of the PLT2 protein, which is one of the key transcription factors for stem cell maintenance (Aida et al., 2004). PLT2 has previously been shown to be regulated by oxidative posttranslational modifications (Shaikhali et al., 2008; Dietz et al., 2010; Licausi et al., 2011, 2013; Waszczak et al., 2014). Moreover, transcriptome analysis did not reveal significant changes in *UPB1* expression upon RGF1 treatment (Yamada et al., 2020). After RGF1 treatment, the expression of five peroxidase genes was elevated, but they were not the targets of UPB1, suggesting that RGF1 regulates meristem size independently of UPB1 (Yamada et al., 2020). Therefore, *RITF1*, induced by the RGF1 peptide, which is secreted from the QC and columella stem cells, regulates PLT2 stability and distribution at the root tip and, thus, controls the meristem size under ROS signaling (Yamada et al., 2020). These data suggest that the RGF1–RITF1–ROS signaling pathway plays a crucial role in the maintenance of the root SCN by regulating PLT stability and distribution. Furthermore, the RGF1 regulatory pathway is independent of the auxin regulatory pathway for PLT (Matsuzaki et al., 2010).

MYB30, in addition to regulating the meristematic zone, is one of the key transcriptional regulators under ROS signaling and has been reported to regulate root cell elongation (Mabuchi et al., 2018). Among the upregulated transcription factors in the “ROS-map,” which is a time-course microarray analysis of *Arabidopsis* root tips treated with H_2_O_2_, *MYB30* showed the most prominent expression induction by H_2_O_2_ in both the meristematic and elongation zones (Mabuchi et al., 2018). MYB30 regulates root growth in response to H_2_O_2_ at the level of cellular elongation in the root tip by upregulating the expression of *LTPG1*, *LTPG2*, and *LTP5* (Mabuchi et al., 2018). *LTPG*s and *LTP* encode lipid transfer proteins that are thought to be the transporter of very-long chain fatty acids (VLCFAs). In fact, root growth inhibition in *ltpg1/2* double mutants showed weak but significant insensitivity to exogenous H_2_O_2_ treatment. This indicates that VLCFA transport to the outside of cells needs to be controlled under ROS signaling for root elongation. Interestingly, MYB30 and its target genes function in not only ROS-dependent root developmental processes in the root tip, but also in plant immune responses toward bacterial elicitors in aerial tissues (Raffaele et al., 2008; Mabuchi et al., 2018). Therefore, the MYB30 regulatory network activated in response to H_2_O_2_ treatment is involved in maintaining the balance between root growth and defense (Mabuchi et al., 2018). In addition, the use of the “ROS-map” revealed another early ROS-responsible transcription factor, *ANAC032*, which is NAC [NAM, no apical meristem; ATAF1/2, *Arabidopsis* transcription activation factor; and CUC2, CUP-shaped cotyledon2 (Souer et al., 1996; Aida et al., 1997; Duval et al., 2002)], as a root growth regulator (Maki et al., 2019). Interestingly, ANAC032 is an upstream transcription factor of the MYB30 regulatory network (Maki et al., 2019). Previous studies suggested that ANAC032 plays important roles in response to abiotic stresses such as high-intensity light, salinity, and oxidation (Mahmood et al., 2016) and is a key mediator between SA- and jasmonic acid-dependent defense signaling (Devi Allu et al., 2016). In the root, ANAC032 plays a dominant role in the transition zone, but not in the apical meristematic zone, and negatively regulates root growth (Maki et al., 2019). These findings suggest that ANAC032 and MYB30 transcriptional cascades are the key regulators of root cell elongation under ROS signaling.

## ROS in Root Stem Cell Identity and Cell Cycle Progression

In the apical meristem, which accumulates mainly O_2_^–^, several systems of SCN maintenance and cell cycle regulation by ROS are available. The *Arabidopsis* prohibitin protein PHB3 regulates the root SCN by restricting the spatial expression of the ethylene response factor (ERF) transcription factors *ERF115*, *ERF114*, and *ERF109* (Kong et al., 2018). The *phb3* mutant accumulated more ROS, leading to an increase in the expression of *ERF115*, *ERF114*, and *ERF109*, which was independent of cell death signaling (Heyman et al., 2013; Kong et al., 2018). In the *phb3* mutant, both the QC-specific transcription factor, *WOX5*, and the QC-specific marker QC184 were strongly downregulated. Furthermore, *PLT1*, *PLT2*, and *SCR*, which are the root SCN-defining transcription factors, were downregulated in the *phb3* mutant. However, the expression of *SHR* was not affected in *phb3*. Interestingly, ERF115, ERF114, and ERF109 directly regulate the expression of peptide hormone precursors, PHYTOSULFOKINE5 (PSK5) and PSK2, which produce sulfonated pentapeptide hormones that regulate cellular dedifferentiation and proliferation (Matsubayashi et al., 1999; Kutschmar et al., 2009; Kong et al., 2018), in parallel with the PLT pathway. In addition, ERF115 controls cell division at the QC and replenishes stem cells by regulating the expression of *PSK5* (Heyman et al., 2013). Thus, the transcriptional network of ERFs controlled through alterations in ROS distribution regulated by PHB3 is essential to maintain root SCN identity and, thus, root development.

The *A. thaliana* P-loop NTPase encoded by APP1 controls ROS homeostasis in the mitochondria of the root apical meristem cells and affects SCN identity (Del Pozo, 2016; Yu et al., 2016). The loss of *APP1* lowers the concentration of O_2_^–^ by upregulating *PER11* and *PER55*, which belong to class III peroxidase family (Yu et al., 2016). In contrast, *APP1* overexpression, as well as elevated ROS levels, promotes cell division at the QC and distal stem cell (DSC) differentiation in the root. In the *app1* mutant, the expression levels of transcription factors such as *WOX5*, *PLT1*, *PLT2*, and *UPB1* and of several cell cycle-related genes were not altered; however, the expression of *SHR* and *SCR* was transcriptionally and translationally reduced (Yu et al., 2016). Therefore, APP1-regulated ROS signaling might regulate cell division at the QC and DSC identity by controlling SHR and SCR functions.

Reactive oxygen species levels also influence cell cycle progression at the transcriptional level. Exogenous H_2_O_2_ treatment affects the expression of G_1_–S and G_2_–M transition-related genes in the meristematic zone (Tsukagoshi, 2012). Repression of cell cycle-related genes by H_2_O_2_ reduces the meristem size, resulting in root growth inhibition. This also supports the role of ROS as signaling molecules that regulate gene expression. In addition, treatment with zeocin, an inducer of DNA double-strand breaks (DSBs), led to the accumulation of H_2_O_2_ in the elongation zone (Chen and Umeda, 2015). DSBs control the coordinated expression of cell cycle-related genes. In plants, DNA lesions such as DSBs or DNA single-strand breaks are sensed by ataxia telangiectasia-mutated (ATM) and ATR and Rad3-related (ATR), respectively (Riballo et al., 2004; Shiotani and Zou, 2009). The downstream transcription factor of these signaling pathways, SUPPRESSOR OF GAMMA RESPONSE 1 (SOG1), governs multiple responses to DNA damage (Yoshiyama et al., 2009, 2013). In the *sog1* mutant, zeocin treatment-induced H_2_O_2_ hardly accumulated in the elongation zone (Chen and Umeda, 2015). SOG1 directly regulates the expression of *FMO1*, which encodes a flavin-containing monooxygenase, and changes in the distribution of H_2_O_2_ upon DNA damage (Chen and Umeda, 2015). Furthermore, SOG1 has been shown to induce directly the expression of *SIAMESE/SIAMESE-RELATED* (*SIM*/*SMR*), *SMR5*, and *SMR7*, which act as cyclin-dependent kinase inhibitors in response to oxidative stress-induced DNA damage (Yi et al., 2014). These data suggest that the SOG1-regulated network plays a central role in the response to DNA damage to facilitate cell cycle progression.

Thus, ROS are considered as one of the important regulators of root meristem activity and cell proliferation by regulating gene expression of key transcription factors for SCN maintenance, such as *PLT*s, *SCR*–*SHR*, and ERFs, as well as cell cycle-related genes.

## ROS in Lateral Root Development

Reactive oxygen species also play an important role in lateral root (LR) development. In the maturation zone, LRs develop from a limited number of pericycle cells called founder cells (Dolan et al., 1993; Casimiro et al., 2003) in the primary root. Founder cells, in response to auxin accumulation at specific sites, undergo anticlinal cell divisions to form an LR primordium (LRP; Malamy and Benfey, 1997; Dubrovsky et al., 2008). Molecular evidence that ROS are involved in LR development was obtained by cell sorting and transcriptomic analysis of S-phase kinase-associated protein 2 (SKP2B)-expressing cells. *SKP2B* encodes an F-box ubiquitin ligase that regulates the division of founder cells (Manzano et al., 2012). Cell sorting was used to identify genes co-expressed with SKP2B-GFP-expressing cells to analyze genes that are specifically expressed during LR development (Manzano et al., 2014), since *SKP2B* is expressed in all stages of the LRP (Manzano et al., 2012). From these cell sorting transcriptomic data sets, numerous genes involved in redox activity (ROS signaling) were identified (Manzano et al., 2012, 2014). Several peroxidase genes were found to be significantly downregulated in *UPB1*-overexpressing plants (Tsukagoshi et al., 2010; Manzano et al., 2014). *UPB1* is expressed in the early stage of LRP development, although its expression seems to be restricted to the peripheral cells of the primordium, and ROS highly accumulate in the emerging LR (Manzano et al., 2014). The *upb1-1* mutant showed a higher number of emerged and mature LRs than those in wild-type plants. In contrast, roots of *UPB1*-overexpressing plants had significantly reduced number of later stages of LRP (Manzano et al., 2014). Thus, ROS signaling involving UPB1-regulated peroxidase genes is important for LR development, especially during LR emergence. In addition, the expression of *UPB1* in the peripheral cells of LRP suggests its role in cell differentiation by repressing peroxidase genes, as noted in the root tip (Tsukagoshi et al., 2010; Manzano et al., 2014).

A new role for MYB36 in LRP development has been revealed (Fernández-Marcos et al., 2017). MYB36 is known to regulate directly and positively the formation of Casparian strips through the expression of *CASP1*, *PER64*, and *ESB1* (Kamiya et al., 2015; Liberman et al., 2015). During LRP development, MYB36 maintains the ROS balance at the LRP boundary in the pericycle cells, which is required for the transition from flat- to dome-shaped primordia by controlling a set of peroxidase genes, *PER9* and *PER64*, and perhaps other peroxidases (Fernández-Marcos et al., 2017). The *myb36* mutant contained more LRP cells along the innermost cell layer than in the wild type; therefore, the lack of *MYB36* produces a flat LRP phenotype. This phenotype is complemented by treatment with potassium iodide, which is a scavenger of H_2_O_2_ (Fernández-Marcos et al., 2017). Furthermore, UPB1 is involved in the regulation of ROS under iron homeostasis. This regulation also controls LR development (Ravet et al., 2009; Briat et al., 2010; Reyt et al., 2015). These results strongly suggest that ROS homeostasis regulated by peroxidases is important for LR development. Peroxidases might be involved in the regulation of cell wall loosening for facilitating the emergence of LRP from overlay cells in the primary root or the reduction of auxin activity by oxidizing IAA (Lagrimini et al., 1997). However, the function of peroxidase genes co-expressing with SKP2B during LR formation is independent of auxin (Manzano et al., 2014).

Respiratory burst oxidase homolog (NADPH oxidase protein)-mediated ROS production also facilitates LR emergence (Orman-Ligeza et al., 2016). AtrbohD and AtrbohF negatively modulate lateral root development by controlling the local generation of superoxide (Orman-Ligeza et al., 2016). The LR density is increased in the double mutants *atrbohD1/F1* and *atrbohD2/F2*, which leads to the production of O_2_^–^, but not of H_2_O_2_, in the maturation zone of the primary root (Li et al., 2015). Thus, the regulation of ROS spatiotemporal accumulation patterns plays a critical role in LR emergence.

Recently, evidence of molecular linkage between auxin and ROS has been reported. Auxin induces H_2_O_2_ accumulation and initiates LR formation (Ma et al., 2014). Auxin upregulates the expression of *RBOH* genes through the transcription factor ROOT HAIR DEFECTIVE SIX-LIKE4 (RSL4; Mangano et al., 2017). Interestingly, *RSL4* is directly regulated by auxin response factors (ARFs; Mangano et al., 2017). Furthermore, a feed-forward regulation among auxin, ROS, and LR development has been recently reported. H_2_O_2_ produced by RBOH, whose expression is induced by RSL4, promotes IAA14 degradation through its downstream product, reactive carbonyl species (RCS; Biswas et al., 2019). These results indicate that a clear molecular interaction exists between ROS and auxin signals that regulate LR development.

## ROS in Root Hair Development

Reactive oxygen species are also involved in the regulation of polar growth, such as in pollen tube and root hair development. Root hairs develop from root epidermal cells and attain a tubular protruding structure in a polar growth manner. In the *Arabidopsis* root, which belongs to type III root hair formation pattern (Datta et al., 2011), the epidermal cells are arranged in files of non-hair cells (N) and hair cells (H; Duckett et al., 1994; Galway et al., 1994). *SCRAMBLED* (*SCM*), a leucine-rich repeat receptor-like kinase, regulates the expression of transcription factors that define the cell fate (Kwak et al., 2005). The molecular mechanisms regulated by transcription factors of N or H cell fate have been extensively studied in *Arabidopsis* (Shibata and Sugimoto, 2019). In N cells, the protein complex of the R2R3-type MYB transcription factor *WEREWOLF* (*WER*; Lee and Schiefelbein, 1999), a bHLH-type transcription factor *GLABLA3* (*GL3*) or its homolog *ENHANCER OF GLABLA3* (*EGL3*; Bernhardt et al., 2003), and the WD repeat protein *TRANSPARENT TESTA GLABLA1* (*TTG1*; Galway et al., 1994) play an important role in suppressing root hair development, thereby enhancing the expression of the homeodomain transcription factor *GLABRA2* (*GL2*), which functions as a negative regulator of root hair differentiation (Di Cristina et al., 1996; Masucci and Schiefelbein, 1996). Conversely, the mobile R3-type MYB transcription factor, *CAPRICE* (*CPC*), plays a key role (Wada et al., 1997) in the development of root hair in H cells. CPC protein moves from N cells to neighboring H cells and binds with GL3/EGL3-TTG1 to form an inactive complex followed by root hair formation through the inhibition of *GL2* expression (Wada et al., 2002; Kurata et al., 2005). Furthermore, several CPC-related R3 Myb proteins, *TRIPTYCON* (*TRY*: Schellmann et al., 2002) and *ENHANCER OF TRY AND CPC1* (*ETC1*; Simon et al., 2007), have been shown to have partially redundant functions (Kirik et al., 2004; Serna, 2008; Tominaga-Wada et al., 2017; Zhou et al., 2020). CPC and its homologs are also required for the induction of the bHLH transcription factor, *ROOT HAIR DEFECTIVE 6* (*RHD6*), which plays key roles in the determination of root hair identity (Masucci and Schiefelbein, 1994; Menand et al., 2007; Shibata and Sugimoto, 2019). The bHLH transcription factor RSL4 regulates root hair growth under the RHD6 regulatory network (Datta et al., 2015; Shibata and Sugimoto, 2019).

Root hair development can be divided into two main stages: root hair initiation and tip growth (Grierson et al., 2014). Rho-type GTPases of plants (ROPs) are required as determinants of the root hair initiation site (Molendijk et al., 2001; Jones et al., 2002; Denninger et al., 2019). ROP guanine nucleotide exchange factor 3 (RopGEF3) recruits ROPs to the future site of hair formation. Before a cell begins to bulge, RopGEF4 is recruited for the positive regulation of tip growth (Denninger et al., 2019). ROOT HAIR DEFECTIVE 2 (RHD2), known as NADPH oxidase or respiratory burst oxidase homolog C (RBOHC), modulates root hair budding (Monshausen et al., 2007; Takeda et al., 2008). The *rhd2* mutant has shorter root hair than the wild type because of the decreased ROS accumulation in the root hair tips. Although RBOHC is the main RBOH in root hair development, RBOHH and RBOHJ are important ROS-producing enzymes in this process (Foreman et al., 2003; Monshausen et al., 2007; Mangano et al., 2017). Of these RBOHs, *RBOHC* and *RBOHJ* are transcriptionally regulated by RSL4 for root tip growth (Mangano et al., 2017). In addition, RSL4 directly regulates the expression of several peroxidase genes (Mangano et al., 2017). ROS accumulation at the root hair tip activates Ca^2+^-permeable channels, which have not yet been identified; this promotes Ca^2+^ influx into the cytoplasm (Foreman et al., 2003; Wu et al., 2010) and modulates cell wall stiffness during rapid hair elongation (Mendrinna and Persson, 2015). The Ca^2+^ gradient observed in wild-type root hair is a continuous gradient in the cytosol, with the highest concentration close to the tip apex (Monshausen et al., 2007; Mendrinna and Persson, 2015). In addition, high levels of cytoplasmic Ca^2+^ trigger ROS production by RBOHs, thereby completing a positive feedback loop during root hair elongation (Takeda et al., 2008). Conversely, PHYTOCHROME AND FLOWERING TIME1 (PFT1)/MED15 subunit of the mediator complex also plays critical roles in root hair morphogenesis (Sundaravelpandian et al., 2013a, b). PFT1 controls the distribution of ROS by activating the gene expression of H_2_O_2_-generating class III peroxidases (Sundaravelpandian et al., 2013a). The *pft1-1* mutant failed to initiate root hair formation and had shorter roots because of reduced expression of PFT1-regulated peroxidase genes (Sundaravelpandian et al., 2013a). Furthermore, inhibition of NADPH oxidase activity by treatment with diphenyleneiodonium also caused defective root hair development by decreasing ROS accumulation at the tip of root hair (Foreman et al., 2003; Lohar et al., 2007). These results suggest the importance of accurate ROS homeostasis at the root hair tips for root hair formation.

Although flavonol levels are low in the epidermis, ROS accumulate in the epidermis. A flavonoid-deficient mutant, *transparent testa 4* (*tt4*), showed increased root hair number and ROS levels in H cells. The *tt4* mutants treated with potassium iodide showed reduced root hair number and ROS accumulation. These results indicate that flavonols act as antioxidants in H cells to control root hair development by modulating ROS accumulation (Gayomba and Muday, 2020).

**FIGURE 1:**
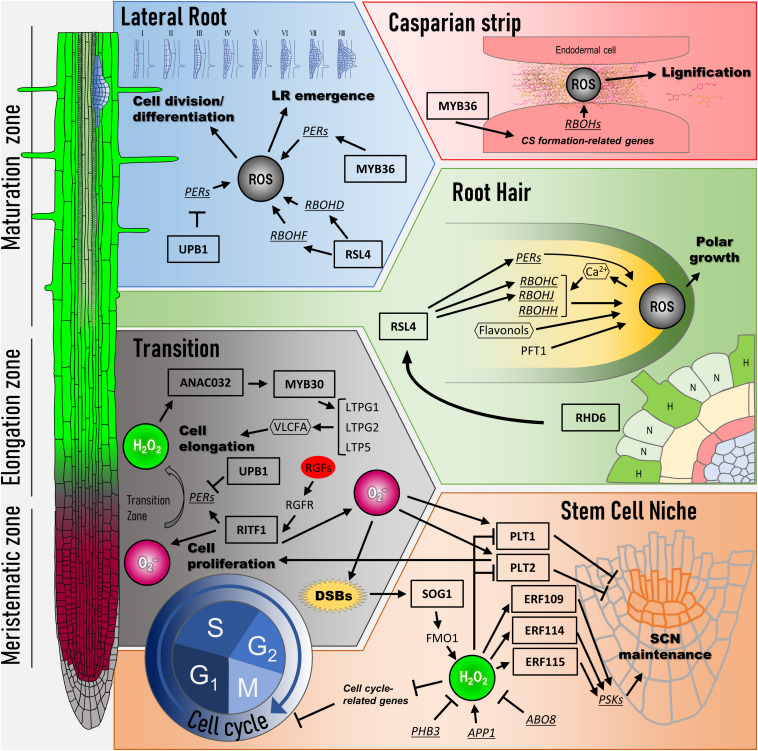
Transcriptional network of ROS signaling for root development. At the left side, three regions in *Arabidopsis* root are shown (meristematic zone, elongation zone, and maturation zone). Color gradient in the primary root indicates the distribution of ROS (red: O_2_^–^ and green: H_2_O_2_). Cell cycle: ROS regulates the expression of G_1_–S as well as G_2_–M transition-related genes. SOG1, a key transcription factor, is activated in response to DNA damage such as double-strand breaks (DSBs). SOG1 directly induces the expression of FMO1, which encodes flavin-containing monooxygenase. H_2_O_2_ level modulated by FMO1 influences the expressions of cell cycle-related genes. Stem cell niche: H_2_O_2_ level is lowered by ABO8, PHB, and RITF1 signaling pathways, whereas APP1 signaling pathway leads to its accumulation. H_2_O_2_ regulates the expression of *ERF109*, *ERF114*, and *ERF115* and represses PLT activity for SCN maintenance. RITF1 signaling pathway positively regulates PLT activity through O_2_^–^ accumulation. Transition: ROS spatial distribution between O_2_^–^ and H_2_O_2_ decides the transition from cell proliferation to cell elongation. UPB1, a key transcription factor, changes ROS spatial distribution by regulating the expression of peroxidases (PERs). RITF1 also controls the meristem size under ROS signaling through PLT2 protein stability. In the elongation zone, ANAC032 and MYB30 regulate the expression of cell elongation-related genes in response to H_2_O_2_. Root hair: Whether epidermal cells can form root hair is determined by gene network (H, hair cells; N, non-hair cells). The RHD6 gene regulatory network begins to bulge at the root hair initiation site in H cells. RSL4, which is under the RHD6 gene regulatory network, controls the expression of *RBOHC*/*RHD2*, *RBOHJ*, and several *PERs* for the root tip growth. A positive feedback loop is formed during hair elongation among Ca^2+^-permeable channels and RBOHs *via* ROS. In addition, ROS distribution in the root hair tip is controlled by PFT1, which regulates the expression of *PERs*. Flavonols contribute to the development of root hair as antioxidants, which modulate ROS accumulation. Lateral root: Lateral root (LR) development is initiated from pericycle cells called founder cells. UPB1 controls LR emergence by regulating the expression of *PERs* at the peripheral cells of LRP. RSL4 regulates the expression of *RBOHs*, followed by ROS production, for facilitating LR emergence. MYB36 in LR development maintains ROS balance at the LRP boundary in the pericycle cells to allow their transition from flat to dome-shaped primordia. Casparian strip: In the endodermal cells, localized ROS in apoplasts induced by RBOHD and RBOHF are utilized for the lignification for the Casparian strip formation. Arrows indicate positive regulation, and blunted lines indicate negative regulation. Ovals, rectangles, and hexagons indicate signal molecules such as plant hormones, transcription factors, and secondary messenger molecules, respectively. PER, peroxidases; DSB, double-strand breaks; SCN, stem cell niche; LR, lateral root; CS, Casparian strip.

The relationship between root development and ROS described so far is shown in Figure 1.

## Crosstalk Between ROS and Other Molecules

The root growth control mechanism involves many plant hormones. In this section, we discuss the interaction of ROS with several other hormone signaling pathways involved in root development, reiterating that ROS act as signaling molecules. When considering signal transduction, determining how each signal interacts is important. In this regard, the crosstalk between ROS and all plant hormones needs to be discussed for understanding the entire complex signal network for root development. However, some of the crosstalks have not been well elucidated at the molecular level, and all of them cannot be discussed at once. In recent years, numerous studies have investigated the crosstalk between ROS and plant hormones during root development. Therefore, we have elaborated on the crosstalks between several important molecules and ROS, which seem to be important for root development.

Even though several studies indicate the molecular linkage between auxin and ROS, the connection between these two signals needs to be further investigated. Although we have already mentioned the molecular linkage between ROS and auxin signaling in LR development, several studies have indicated the independence of these two signals. Thus, we need to elucidate how the distribution of auxin is regulated by ROS, and how ROS regulate the stability of auxin signal regulators at the molecular level. A recent study provided evidence for the relationship between auxin distribution and ROS action in the root tip. The exogenous application of H_2_O_2_ led to auxin accumulation in the root apical meristem, along with a decrease in the abundance of PIN auxin efflux carriers (Zwiewka et al., 2019). In particular, H_2_O_2_ interferes with the intracellular trafficking of PIN2, leading to the decrease in PIN2 protein levels in the plasma membrane of root epidermal cells (Zwiewka et al., 2019). This affects root meristem size by altering the auxin maxima. However, this alteration in PIN2 trafficking is an early event in response to oxidative stress. Studies need to assess the long-term responses for revealing the entire crosstalk between ROS and auxin distribution. With regard to protein degradation, RCS, which is a downstream target of ROS in auxin signaling (please also see section “ROS in Lateral Root Development”), regulates IAA14 protein stability (Biswas et al., 2019). However, the mechanism by which RCS controls IAA stability has not yet been studied (Biswas et al., 2019). Determining whether TIR1 or E3 ligase in the SCF–TIR1 complex is activated by ROS or RCS is important to better understand the molecular mechanism that regulates the ROS–RCS–auxin signal at the posttranscriptional level.

The crosstalk between cytokinin and ROS is also important because cytokinins are closely associated with auxin for regulating root growth. The overproduction of endogenous cytokinin by the overexpression of the cytokinin biosynthetic gene, *ADENOSINE PHOSPHATE-ISOPENTENYL TRANSFERASE 8* (*AtIPT8*), in *Arabidopsis* roots does not affect ROS levels under normal conditions. However, *AtIPT8* overexpressors accumulate more ROS than the wild type after NaCl treatment. Under these conditions, *AtIPT8* overexpressors showed a short root phenotype. Moreover, several NADPH oxidases, which are known as ROS-producing enzymes, are upregulated after NaCl treatment in *AtIPT8* overexpressors. Conversely, ROS scavenging-related genes were downregulated after NaCl treatment in the *AtIPT8* overexpressors (Wang et al., 2015). According to that study, although only cytokinin overproduction cannot lead to the accumulation of ROS in the roots, cytokinins might enhance the salinity stress that inhibits root growth by modulating ROS accumulation. However, a study on root phototropism elucidated the relationship between cytokinins, ROS, and flavonols. In that study, flavonols were found to be regulators of root phototropism by transcriptomic and metabolomic profile analysis (Silva-Navas et al., 2016). In fact, flavonols accumulate in the transition zone at the root tip and reduce cell proliferation by scavenging superoxide anions (Silva-Navas et al., 2016). Cytokinins induce flavonol biosynthesis through SHORT HYPOCOTYL 2 (SHY2), which is a transcription factor limiting meristem size under cytokinin signaling (Dello Ioio et al., 2008). Even though this result indicates that ROS levels are not directly controlled by cytokinins, ROS downstream of cytokinins might control the transition between cell proliferation and differentiation. Interestingly, H_2_O_2_ accumulation regulated by UPB1 also controls flavonol content at the root tip (Silva-Navas et al., 2016). These results indicate the existence of a complex crosstalk between cytokinins and ROS signaling during the regulation of plant root growth.

In addition to auxin and cytokinin, BRs are important plant hormones that regulate many aspects of plant growth and development. The molecular mechanism between UPB1 and BR that regulates root growth has recently been elucidated. In the BR signaling pathway, the phosphorylation of signal component proteins is crucial. BR signals are perceived by the receptor kinase BRASSINOSTEROID-INSENSITIVE 1 (BRI1). BRI1 interacts with coreceptors, BRI1-ASSOCIATED RECEPTOR KINASE 1 (BAK1) and SOMATIC EMBRYOGENESISRECEPTOR KINASEs (SERKs), to transmit the signals downstream by protein phosphorylation (Shang et al., 2016; Wang H. et al., 2018). One of the BRI1 downstream kinases, BRASSINOSTEROID INSENSITIVE 2 (BIN2), interacts with UPB1 and phosphorylates UPB1 (Li et al., 2020). Phosphorylated UPB1 interacts with other BR signal-related bHLH proteins, paclobutrazol-resistant proteins 2 and 3 (PRE2/3), and controls downstream gene expression. Moreover, a transcription factor involving BR signaling, BRI1-EMS-SUPRESSOR 1 (BES1), directly regulates *UPB1* expression (Li et al., 2020). Because of these transcriptional regulation and protein interactions, root growth, especially root meristem development, is regulated. These results strongly indicate that two signaling pathways between ROS and BR are connected through UPB1–BIN2 interactions.

Abscisic acid is known to be the key regulator of both abiotic and biotic stresses (Nakashima et al., 2014). As for root growth, ABA affects auxin distribution and PLT protein stability through the production of ROS in the mitochondria of the root tips (Yang et al., 2014). A mutant, *aba-overly sensitive 8-1* (*abo8-1*), exhibits retarded growth and hypersensitivity to ABA. *ABO8*, which encodes pentatricopeptide repeat protein, is highly expressed in the root tips and LRP and regulates the splicing of mitochondrial complex I NAD4 intron 3. The *abo8-1* mutant shows excessive accumulation of ROS because of the incomplete mitochondrial electron transport chain of complex I. High accumulation of ROS in *abo8-1* reduces the expression of *PLT* genes and root meristem activity, thereby altering auxin distribution (Yang et al., 2014). These results suggest that appropriate ROS levels in the mitochondria are crucial mediators of root SCN maintenance and root growth through auxin distribution. This also indicates the existence of a crosstalk among ROS–ABA–auxin for the regulation of root meristem size. Moreover, the MYB30 regulatory gene network for root elongation is regulated by ABA. ABA induces *MYB30* expression in the root, but ROS accumulation levels after ABA treatment in the roots are not altered in both wild type and *myb30* mutants (Sakaoka et al., 2018). These results indicate that MYB30 acts as a hub between ROS and ABA signaling to regulate root cell elongation.

Reactive oxygen species are also known as signaling molecules in plant immune responses. In the aerial part, ROS are rapidly produced and accumulated by pathogen attack, which is called oxidative burst (Peleg-Grossman et al., 2012). This leads to defense against the attacking pathogens as well as the modification of the cell wall to become stiffened (Denness et al., 2011). The burst is induced by microbe-associated molecular patterns (MAMPs; Albert, 2013). Exogenous treatment with Flg22, which is one of the MAMPs, increases ROS levels, followed by a decrease in root development (Gómez-Gómez et al., 1999). This phenomenon indicates a molecular linkage between ROS and biotic stresses. MYB30 is known to activate the hyperresponse after the oxidative burst (Raffaele et al., 2008). Flg22 treatment shortened mature cell length in the wild-type root, but this phenotype was alleviated in *myb30* mutants (Mabuchi et al., 2018). Moreover, H_2_O_2_ levels increased upon Flg22 treatment in both wild type and *myb30* mutants to the same extent (Mabuchi et al., 2018), indicating that MYB30 regulates root length by increasing ROS levels caused by Flg22 treatment, not because of ROS biosynthesis but because of a defect in signaling downstream of ROS biosynthesis. Indeed, the expression levels of *MYB30* and several target genes such as *LTPG2* and *LTP5* were induced by Flg22 treatment (Mabuchi et al., 2018). *FLAGELLIN SENSING 2* (*FLS2*), which is a receptor kinase of Flg22, is expressed in the vasculature of the root elongation zone (Gómez-Gómez et al., 1999; Beck et al., 2014) and is upregulated in almost all cell files by Flg22 and H_2_O_2_ treatments (Beck et al., 2014). Moreover, *the fls2* mutant showed insensitivity to Flg22 treatment, which regulated root elongation. These results provide excellent evidence that ROS and MYB30 induced by ROS are important for the connection between root growth and biotic stresses such as plant immune response.

With regard to plant immune responses, SA is a crucial signaling molecule (Zhou and Zhang, 2020), and SA is related with ROS in various stress responses (Herrera-Vásquez et al., 2015). SA signals are received by the receptor proteins NON-EXPRESSOR OF PR GENES 1 (NPR1) and its paralogs NPR3 and NPR4 (Ding et al., 2018), which regulate the expression of downstream SA-dependent genes. Similar to that in shoot parts, SA treatment leads to the accumulation of ROS in the root tip through the upregulation of the gene expression of *RBOHD* and *RBOHF*, and the ROS control cell activity at the QC through PLTs and WOX5 regulation (Wang et al., 2021). However, the upregulation of *RBOHD* and *RBOHF* in root tips by SA treatment is not observed in the *npr1* mutant and *npr3/4* double mutants. This indicates that the regulation of the expression of *RBOHD* and *RBOHF* by SA is NPR dependent. Therefore, a part of the ROS accumulation upon SA treatment related to SCN maintenance is regulated by the SA–NPR regulatory system. Auxin and ethylene are also involved in this regulation *via* PLT and ERFs (Kong et al., 2018; Wang et al., 2021). Although these studies focused on SCN maintenance, considering that Flg22 activates SA signals is important, because SA as well as Flg22 may regulate cell elongation by interacting with ROS signaling.

## Discussion

**FIGURE 2:**
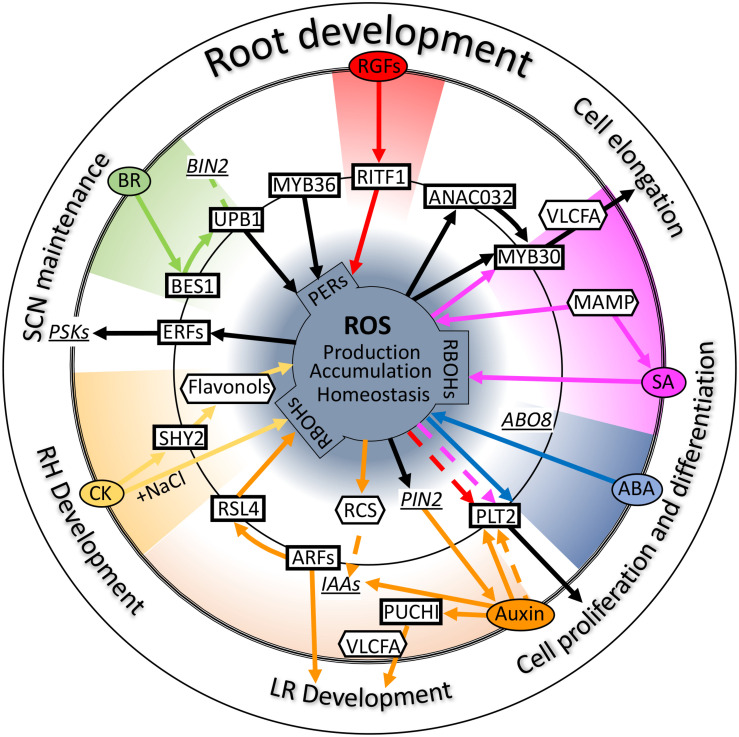
Crosstalk between reactive oxygen species (ROS) and plant hormones. Schematic diagram of the crosstalk of several hormones and ROS signals centered on some key transcription factors involved in root development. Ovals, rectangles, and hexagons indicate signal molecules such as plant hormones, transcription factors, and secondary messenger molecules, respectively. Underlined italicized letter indicate genes involved in these signals as important intermediators. Solid lines refer to “transcriptional regulation,” and dotted lines indicate “regulation at the protein level.” Black lines indicate direct regulation by ROS or regulation of ROS homeostasis. Colored lines indicate each signal transduction. Please see the main text for further details. Auxin (orange); ABA, abscisic acid (blue); BR, brassinosteroid (green); CK, cytokinin (yellow); SA, salicylic acid (purple); RGFs, root meristem growth factors (red); PERs, peroxidases; PSKs, phytosulfokines; RCS, reactive carbonyl species; LR, lateral root; RH, root hair; SCN, stem cell niche.

In this review, we introduce and discuss the functions of ROS as a root growth regulator. Modifying biological substances by the chemical activity of ROS itself is important, but ROS are known to play a more important role as a signaling molecule through gene expression regulation in a broad range of aspects of root development and stress responses (Figure 2). Indeed, ROS regulate the proliferation of cells, determination of cell identity, differentiation of root cells, and adaptation to even more biotic and abiotic stress. Considering the function of ROS, elucidation of all aspects of ROS signaling would provide information regarding the control of root development and stress response. For this, the perception of ROS signals, that is, the starting point of signal transduction, needs to be determined. Many studies have indicated that ROS are generated by NADPH oxidases localized on the plasma membrane. However, ROS such as superoxide and hydrogen peroxide are produced in the apoplast. Because of the chemical features of ROS, they cannot permeate the plasma membrane. For signal transduction with gene expression regulation, ROS generated by NADPH oxidase should be transported into cells. Although H_2_O_2_ is known to be transported from the apoplast to the cytosol *via* aquaporins (Waszczak et al., 2018), H_2_O_2_ sensors on the plasma membrane were not identified for a long time. Recently, an LRR receptor kinase, hydrogen peroxide-induced Ca^2+^ 1 (HPCA1), was identified as a H_2_O_2_ sensor that regulates Ca^2+^ channels on the plasma membrane (Wu et al., 2020). HPCA1 activates Ca^2+^ channels upon binding to H_2_O_2_ and regulates stomatal closure (Wu et al., 2020). To our knowledge, this was the first report of a cell surface sensor for H_2_O_2_ in plants. In this case, H_2_O_2_ transduces the signal *via* Ca^2+^ influx, followed by an increase in Ca^2+^ concentration in the cell. However, H_2_O_2_ does not act as the primary signal for regulating gene expression. Although whether HPCA1 is a H_2_O_2_ signal receptor for root development is not known, HPCA1 can be used to explore how H_2_O_2_ signals are transduced from the outside of cells. If HPCA1 or its homologs function in the roots to activate Ca^2+^ channels, the role of Ca^2+^ in root hair development could be elucidated. Furthermore, whether any H_2_O_2_ sensor other than HPCA1 is present in plant cells is not yet known. Identification of such a sensor or receptors would lead to the elucidation of the ROS regulatory gene expression network; nonetheless, the function of some transcription factors in yeast and bacteria has been shown to be modulated in the presence of H_2_O_2_ to modulate their function (Zheng et al., 1998).

In addition to the perception of ROS signals in a cell, cell-to-cell ROS signal transduction is also important for understanding ROS function. ROS and Ca^2+^ induced by local stress have been shown to activate a calcium-dependent autopropagating wave of ROS. The ROS wave spreads to the entire plant and causes ROS-specific responses in distant organs (Suzuki et al., 2013). In this case, ROS itself and several secondary signal molecules such as calcium ions and plasma membrane-electric potential form rapid and distant waves together (Gilroy et al., 2014). This wave is triggered by abiotic stresses such as heat and light (Suzuki et al., 2013). Whether the ROS wave also regulates plant root development is not yet known. According to the evidence of the crosstalk of ROS with other hormone signaling pathways, a regulatory mechanism that transduces the ROS signal to the adjacent cells might exist to regulate cell differentiation by the ROS wave. If appropriate ROS fluorescent markers are available that respond rapidly and ROS-specifically, such as R2D2 for auxin indicator (Liao et al., 2015), ROS wave for root development can be elucidated using live imaging.

We also discussed the crosstalk between ROS and several other molecules, mainly plant hormones involved in root development. In several cases, secondary molecules that connect ROS and hormone signals are important, for example, RCS in auxin, flavonols in cytokinin, and calcium ions in root hair development and ROS wave. These secondary molecules play important roles as hubs in the signal crosstalk. Other secondary molecules involved between ROS and root development include VLCFA. *MYB30* overexpression upregulates VLCFA synthesis genes in the leaves and roots (Raffaele et al., 2008; Mabuchi et al., 2018). This indicates that VLCFA is induced by H_2_O_2_ because *MYB30* is an H_2_O_2_-inducible gene. In fact, in the wild-type root, the expression of VLCFA synthesis genes was upregulated upon H_2_O_2_ treatment (Mabuchi et al., 2018). Independent of these studies, VLCFA has been shown to be an important regulator of lateral root emergence (Trinh et al., 2019). VLCFA synthetic genes are expressed by the AP2 family transcription factor, PUCHI, in developing LRP, and mutants of these genes show lateral root defects (Trinh et al., 2019). This result indicates that VLCFA acts as a regulator of lateral root development. Considering the accumulation pattern of ROS in the LRP, a large amount of ROS accumulated in the LR (Manzano et al., 2012). These two studies strongly indicate the possibility that VLCFA acts as a secondary molecule for LR development under ROS signaling. In addition, auxin leads to the accumulation of ROS (Gayomba and Muday, 2020) and is presumed to accumulate in the LRP, causing the accumulation of ROS. This possibility needs to be confirmed experimentally, but the molecular relationship among auxin–ROS–VLCFA is considerably interesting for lateral root development.

Another interesting secondary molecule under ROS signaling is microRNA. Several studies have identified many microRNAs, including microRNA160, 165/166, and 393A, which respond to several abiotic stresses such as oxidative, cold, salt, and UV-B (Iyer et al., 2012; Barciszewska-Pacak et al., 2015). According to the functions of microRNA160, which targets ARF10, ARF16, and ARF17 (Khan et al., 2011), and microRNA393A, which targets TIR1 and AFB2 (Iglesias et al., 2014), ROS-responsive microRNAs can act as secondary molecules connecting ROS and auxin signals. microRNA165/166 suppresses class III homeodomain leucine zipper (HD-ZIP III), primarily PHABULOSA (PHB), which regulates protoxylem differentiation (Carlsbecker et al., 2010). Moreover, microRNA165/166 functions non-cell-autonomously in the root (Carlsbecker et al., 2010; Miyashima et al., 2011). According to their nature, microRNAs that respond to ROS may play a role as secondary molecules for cell–cell communication in root development and stress responses.

Although, in this review, we only focused on ROS function in root development, they may also be important for all aspects of plant growth. ROS play a pivotal role in plant root development and are the connection hub of other signaling pathways. Moreover, the participation of secondary molecules downstream of ROS has also been shown to be important for ROS signal transduction. However, many missing links exist in ROS signaling. With the development of latest technology, further research can be performed to reveal the mode of signal transduction at the single-cell level by using single-cell omics. In addition, the dynamics of inter- and intracellular ROS would be revealed by live imaging performed using super-resolution microscopes. These findings suggest that new technologies for plant growth control can be developed by targeting relatively common molecules such as ROS.

## Author Contributions

HT revised, guided, and improved the manuscript. Both authors carefully revised and edited the manuscript, replotted the figures, and drafted the manuscript.

## Conflict of Interest

The authors declare that the research was conducted in the absence of any commercial or financial relationships that could be construed as a potential conflict of interest.

## Abbreviations

ABA: abscisic acid
ARFs: auxin response factors
bHLH: basic helix-loop-helix
BRs: brassinosteroids
CASPs: Casparian strip domain proteins
DSBs: double-strand breaks
ERF: ethylene response factor
FLS2: *FLAGELLIN SENSING 2*
LR: lateral root
LRP: LR primordium
MAMPs: microbe-associated molecular patterns
NPR1, NON-EXPRESSOR OF PR GENES 1. PLT: PLETHORA
PSK5: PHYTOSULFOKINE 5
QC: quiescent center
RBOH proteins: RESPIRATORY BURST OXIDASE HOMOLOG proteins
RCS: reactive carbonyl species
RGF1: root meristem growth factor 1
*RHD6*: *ROOT HAIR DEFECTIVE 6*
RITF1: RGF1 INDUCIBLE TRANSCRIPTION FACTOR 1
ROPs: Rho-type GTPases of plants
ROS: reactive oxygen species
RSL4: ROOT HAIR DEFECTIVE SIX-LIKE 4
SA: salicylic acid
SCN: stem cell niche
SCR: SCARECROW
SHR: SHORT ROOT
SKP2B: S-phase kinase-associated protein 2
SOD: superoxide dismutase
SOG1: SUPPRESSOR OF GAMMA RESPONSE 1
VLCFAs: very-long chain fatty acids
WOX5: WUSCHEL-related homeobox 5.
